# Mucosal SIV Vaccines Comprising Inactivated Virus Particles and Bacterial Adjuvants Induce CD8^+^ T-Regulatory Cells that Suppress SIV-Positive CD4^+^ T-Cell Activation and Prevent SIV Infection in the Macaque Model

**DOI:** 10.3389/fimmu.2014.00297

**Published:** 2014-06-30

**Authors:** Jean-Marie Andrieu, Song Chen, Chunhui Lai, Weizhong Guo, Wei Lu

**Affiliations:** ^1^Institut de Recherches sur les Vaccins et l’Immunothérapie des Cancers et du SIDA, Centre Universitaire des Saints Peres, Université de Paris-Descartes, Paris, France; ^2^Tropical Medicine Institute, Guangzhou University of Chinese Medicine, Guangzhou, China

**Keywords:** inactivated SIV, bacterial adjuvants, BCG, lactobacilli, SIV replication, SIV vaccine, HIV vaccine, HIV tolerogenic vaccine

## Abstract

A new paradigm of mucosal vaccination against human immunodeficiency virus (HIV) infection has been investigated in the macaque model. A vaccine consisting of inactivated simian immunodeficiency virus (SIV)mac239 particles together with a living bacterial adjuvant (either the Calmette and Guerin bacillus, *Lactobacillus plantarum* or *Lactobacillus rhamnosus*) was administered to macaques via the vaginal or oral/intragastric route. In contrast to all established human and veterinary vaccines, these three vaccine regimens did not elicit SIV-specific antibodies nor cytotoxic T-lymphocytes but induced a previously unrecognized population of non-cytolytic MHCIb/E-restricted CD8^+^ T-regulatory cells that suppressed the activation of SIV-positive CD4^+^ T-lymphocytes. SIV reverse transcription was thereby blocked in inactivated CD4^+^ T-cells; the initial burst of virus replication was prevented and the vaccinated macaques were protected from a challenge infection. For 3–14 months after intragastric immunization, 24 macaques were challenged intrarectally with a high dose of SIVmac239 or with the heterologous strain SIV B670 (both strains grown on macaques PBMC). Twenty-three of these animals were found to be protected for up to 48 months while all 24 control macaques became infected. This protective effect against SIV challenge together with the concomitant identification of a robust *ex vivo* correlate of protection suggests a new approach for developing an HIV vaccine in humans. The induction of this new class of CD8^+^ T-regulatory cells could also possibly be used therapeutically for suppressing HIV replication in infected patients and this novel tolerogenic vaccine paradigm may have potential applications for treating a wide range of immune disorders and is likely to may have profound implications across immunology generally.

## Introduction

Since the first vaccination against the small pox virus by Edward Jenner in 1796, all efficient vaccines against a viral infection have been shown to elicit virus-specific neutralizing antibodies and sometimes also cytotoxic T-lymphocytes (CTL) that prevent virus infection or eradicate the virus rapidly after it enters the body (1). So far, however, this process seems not to work for the human immunodeficiency virus (HIV) type 1 infection since, despite the tremendous advances in immunology and molecular biology accomplished since HIV discovery in 1983 (2), results of vaccine trials against HIV have indeed remained extremely poor (3). Only one trial out of more than one hundred showed a modest and short-lasting protection (4) while all the others did not induce any protective immunity (antibodies or CTL) against the virus including sadly the latest three randomized vaccine trials where more volunteers were infected in the vaccine arm than in the placebo arm (5–7); combining data from the three studies, there was an overall hazard ratio of 1.33 (*P* < 0.01) associated with vaccination (8).

For the last 30 years, our group tried to suppress HIV replication and fight HIV infection by pursuing several alternative immunological approaches. As early as 1986, we speculated that T4-cell activation (whether triggered by the virus, viral proteins, or by any other stimulus) was an absolute requirement for significant viral replication (9). This suspicion, which is now commonly accepted by scientists who study the pathogenesis of simian immunodeficiency virus (SIV) in macaques and of HIV in humans (8, 10, 11) suggested to us that it might be possible to inhibit virus replication by suppressing CD4^+^ T-cell activation (9). We tested the possibility that viral replication might be controlled by administering the immunosuppressive drugs cyclosporine or prednisolone to infected patients. Both drugs were found to stabilize or increase CD4^+^ T-cell counts in HIV-infected patients (12–15), probably by preventing activation-induced apoptosis of non-infected CD4^+^ T-cells (16), but they did not control HIV-1 replication.

Since it was not possible to control viral replication by systemic non-specific immune suppression, we then explored the capacity of dendritic cells loaded with *ex vivo*-inactivated virus to stimulate HIV-specific cellular immunity (17, 18). Using this approach, we were able to demonstrate that a therapeutic vaccine based on inactivated HIV-loaded dendritic cells had a favorable impact on HIV replication (19), a finding that was recently confirmed in HIV-infected patients who had interrupted their antiviral therapy (20). However, we did not pursue this project because the preparation of dendritic cell-based vaccine was cumbersome and expensive and was incompatible with a large scale use.

Instead of activating dendritic cells by loading them *ex vivo* with inactivated HIV, we then investigated possibility of developing a prophylactic anti-SIV vaccine by directly stimulating mucosal dendritic cells *in vivo*. We describe here the attempts we made during the last 10 years to develop prophylactic mucosal vaccines using inactivated SIV mac239 (iSIV) adjuvanted by non-pathogenic living bacteria potentially able to cooperate with mucosal dendritic cells. These experiments were performed in macaques of Chinese origin, which best mimics HIV infection in humans (21, 22).

## Materials and Methods

### Animals

Colony-bred rhesus macaques (*Macaca mulatta*) of Chinese origin were housed in accordance with the regulations of the National Institutes of Health “Guide for the Care and Use of Laboratory Animals.” The committee for animal studies of the Tropical Medicine Institute, Guangzhou, University of Chinese Medicine, has approved the study and confirmed that it has been done appropriately. All animals were in good health, 2–4 years old, weighed 4–6 kg, and were seronegative for SIV, simian retrovirus, simian T-cells lymphotropic virus 1, hepatitis B virus, and Herpes virus simiae.

### Vaccine preparation

The vaccines used throughout this study were a mixture of two components: the iSIV and a bacterial adjuvant, which was either the Bacillus of Calmette & Guerin (BCG), the *Lactobacillus plantarum* (LP), or the *Lactobacillus rhamnosus* (LR).

#### Bacterial preparations

Three bacterial preparations were used: the BCG, the LP, and the LR. The BCG (strain SSI 1331, Sanofi-Pasteur) was used at a final dose of 5 × 10^6^ or 7.5 × 10^7^ cfu for each (intravaginal or intragastrical) vaccination or boost. Each dose of vaccine was freshly prepared in 1 mL of RPMI. The LP (ATCC8014) was cultivated at 37°C in MRS medium with a rotation rate of 200 rpm until reaching a final LP concentration of around 10^10^ cfu/ml (i.e., an optical density of 1.0 at 600 nm). The LR (lcr35, Probionov, 15000 Aurillac, France) was received in lyophilized form at a concentration of around 1 × 10^11^ cfu/g.

#### Inactivated virus preparation

The virus production was performed in CEM174 cells inoculated with SIVmac239 [see Lu et al. (23)]. Culture supernatants were collected at peak viral production. In order to check what could be the simplest and safest inactivation system, we tested three different modalities: when associated with the BCG, the virus was inactivated with 250 μM aldrithiol-2 (AT-2) in the same manner as we did previously (18); when associated with LP, the virus was inactivated with AT-2 and then by heat (56°C for 30 min) [see Lu et al. (23)]; finally, when associated with LR, the virus was inactivated in the simplest as possible manner, by heat (56°C for 30 min) twice at 30 min-interval. The iSIV was inoculated to CEM174 cells to verify the 100% inhibition of viral infectivity.

### Delivery of the vaccines

Monkeys were anesthetized with tiletamine hydrochloride and intramuscular zolazepam (0.7 mg/kg) (after having fasted overnight for those which were immunized via the intragastric route).

#### Intravaginal immunization with iSIV + BCG

Monkeys were administered intravaginally 5 ml of a vaccine comprising 10^9^ particles of iSIV and 5 × 10^6^ cfu of BCG (strain SSI 1331). A booster intravaginal immunization with the same dose was applied 2 months later. Control animals remained naïve until challenge.

#### Intragastric immunization with iSIV + BCG

Monkeys after having fasted overnight were anesthetized. They were administrated intragastrically with 25 ml of PBS buffer and half an hour later with 5 ml of a viral–bacterial preparation containing 10^9^ copies/ml of iSIV and 1.5 × 10^7^ cfu/ml of BCG in maltodextrin (20%) solution. A booster intravaginal immunization with the same dose was applied 2 months later. Control animals received the same doses of BCG alone according to the same protocol.

#### Intragastric immunization with iSIV + LP or LR

The macaques were administrated intragastrically 30 ml of a first preparation containing 4 × 10^7^ copies/ml of iSIV and 3 × 10^9^ cfu/ml of LP or LR in maltodextrin (20%) solution. Monkeys then received intragastrically 25 ml of the same preparation each 30 min for 3 h; the same protocol was performed over five consecutive days. Overall, each macaque received a total of 3.5 × 10^10^ copies of iSIV and 2.6 × 10^12^ cfu of LP or LR. Control animals received the same doses of LP alone, LR alone, or iSIV according to the same protocol.

Overall, 37 macaques were vaccinated by one of the above-mentioned protocols and 45 animals served as control.

### SIV suppression assay: *ex vivo* antiviral activity of vaccine-induced CD8^+^ T-cells

Autologous CD4^+^ T-cells from each animal purified by magnetic positive-labeling (MicroBeads, Miltenyi Biotec) were acutely infected with SIVmac239 (10^−3^ multiplicity of infection) in the presence or the absence of magnetically purified CD8^+^ T-cells at a CD4/CD8 ratio of 1:3 and then stimulated with staphylococcal enterotoxin B (SEB) and anti-CD3/anti-CD28 antibodies for 16 h. After washing, the cells were cultivated in quadruplicates in 96-well plates. Cultures were maintained in a final volume of 200 μl per well of RPMI 1640 medium containing 100 IU of human rIL2 (Roche Diagnostics GmbH, Mannheim, Germany) for 5 days. Viral loads were measured by a real-time RT-PCR in culture supernatants collected at day 5 [see Lu et al. (23)]. *Ex vivo* antiviral activity was the ratio of geometric means of viral concentration in the culture supernatants from the infected CD4^+^ target cells only over the geometric means of viral concentration in the supernatants from the mixed CD8^+^ and CD4^+^ T-cells.

### Viral challenges

For 3–14 months after the administration of the vaccine or the controls, vaccinated and control animals were inoculated intravenously with 50 MID_100_ (200 TCID_50_) or intrarectally with 2,500 MID_100_ (100,000 TCID_50_) of pathogenic SIVmac239 (cultivated on macaques PBMC). Monkeys were rechallenged by intravenous or intrarectal route with the same high doses of infectious SIVmac239. Eight macaques were intrarectally rechallenged with 100,000 TCID_50_ of pathogenic SIVB670 (a distinct strain of SIV provided by F. Villinger, Emory University School of Medicine, Atlanta, Georgia). SIVB670 was propagated *in vitro* using macaque PBMC and the first passages of the original viral stocks were used for challenge.

### Other methods

For details on other methods used in this study (MHC class I typing, assay for antibody responses to SIV, flow cytometry, SIV-specific cell proliferation assay, SIV-specific ELISPOT assay, T-cell suppression assay, T-cell cytotoxicity assay, viral loads measurements, depletion of CD8^+^ T-cells *in vivo*, and statistical analysis), see Section “Experimental Procedures” in Lu et al. (23).

## Results

### Mucosal vaccination with inactivated SIV and BCG

We first tested a vaccine including iSIV as immunogen and the BCG as a bacterial adjuvant (24). We chose the BCG because it had been shown to interact with dendritic cells (25, 26) as well as to stimulate cellular immunity in some types of cancers (27, 28). Moreover, BCG had been given orally to several millions of people in Brazil until 1974 without serious side effects (29).

#### Intravaginal immunization followed by intravenous challenge

**Figure 1:**
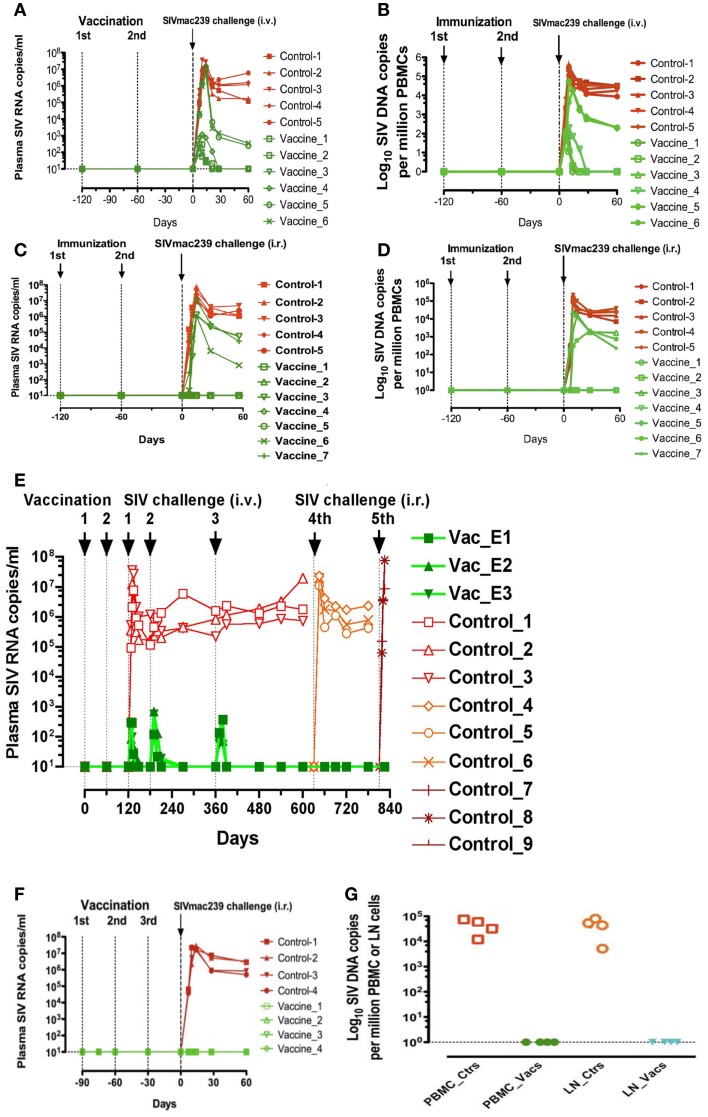
**Plasma viral loads and PBMC and lymph nodes proviral loads of rhesus macaques immunized with inactivated SIV and BCG**. **(A,B)** Plasma viral loads **(A)** and PBMC proviral loads **(B)** following a challenge performed intravenously with SIVmac239 (200 TCID50) in six macaques intravaginally immunized with iSIV and BCG and five control monkeys; **(C,D)** plasma viral loads **(C)** and PBMC proviral loads **(D)** following a challenge performed intrarectally with SIVmac239 (100,000 TCID50) in seven macaques intravaginally immunized with inactivated SIV and BCG; **(E)** Plasma viral loads of three macaques intravaginally immunized with inactivated SIV and BCG following three intravenous and two intrarectal SIVmac239 challenges; **(F,G)** Plasma viral loads **(F)** and PBMC and lymph nodes proviral loads **(G)** following a challenge performed intrarectally with SIVmac239 (100,000 TCID50) in four macaques intragastrically immunized with inactivated SIV and BCG and four control monkeys immunized with inactivated BCG alone (ctrs: control, vacs: vaccinated, LN: lymph nodes).

Six macaques were administered intravaginally 5 ml of a vaccine comprising 10^9^ particles of iSIV and 5 × 10^6^ cfu of BCG (strain SSI 1331). A booster intravaginal immunization was applied 2 months later. Two months later (i.e., 4 months after the initial vaccination), the six animals were challenged by intravenous route with 200 TCID_50_ of SIVmac239. Simultaneously five vaccine-free control animals were also challenged with the same dose of SIVmac239. Al control macaques showed a typical primary infection with plasma viral loads peaking at 10^6^–10^7^ viral copies/ml between days 10 and 14 post-challenge and then remaining high (>10^5^ copies/ml) over the next 60 days. They also developed high levels of PBMC SIV DNA and became seropositive with high titers of SIV antibodies. Among the six animals that received the intravaginal vaccine, plasma viral RNA loads of two of them, after the usual peak (>10^6^ copies/ml), decreased to lower set-point (<10^3^ copies/ml) than normal whereas their SIV DNA remained ≤1,000 copies/10^6^ in PBMC or lymph node cells. More surprisingly, the four remaining macaques showed very low viral RNA and DNA peaks (≤10^3^ SIV RNA copies/ml and <10^3^ SIV DNA copies//10^6^ PBMC) by days 10–14, which dropped down to undetectable/hardly detectable levels thereafter (Figures 1A,B).

#### Intravaginal immunization followed by intrarectal challenge

Seven new macaques were then administered intravaginally the same vaccine with the same boosts while five controls animals remained vaccine-free. Four months later, the 12 animals were challenged via the intrarectal route with 100,000 TCID_50_ of SIVmac239. The five control animals showed the same typical primary infection as described above. Moreover, three out of the seven vaccinated animals showed also a primary infection resembling the usual one but with viral RNA and DNA load set-points significantly lower than those observed in control animals. The new information was that four out of the seven vaccinated macaques remained with undetectable levels of plasma SIV RNA (<10 SIV RNA copies/ml) and PBMC proviral DNA (<1 SIV DNA copies/10^6^ PBMC) over the 60 days post-challenge (Figures 1C,D).

#### Protection against repeated intravenous or intrarectal challenges after intravaginal immunization

Among the four animals with undetectable viral loads following intravenous challenge, three were tested for their long-term capacity to fight new SIV challenges. For 2 and 8 months after their initial intravenous challenge (i.e., 6 and 12 months after their immunization), these three monkeys received a second and a third intravenous challenge with the same dose of SIV239 (200 TCID_50_). After each of these viral challenges, similar low plasma RNA SIV peaks were observed at day 10 but by day 30, viral loads of all three macaques had dropped down to undetectable levels. For 21 and 27 months after initial vaccination, the three macaques were further challenged with SIVmac239, this time via the intrarectal route (100,000 TCID_50_) and the three animals showed again no detectable plasma virus (<10 SIV RNA copies/ml) while their PBMC SIV DNA levels remained at the limit of positivity. Overall, viral replication of these three vaccinated macaques remained stably suppressed as long as we tested it (28 months) in spite of five SIV239 challenges (Figure 1E).

#### Oral (intragastric) immunization followed by intrarectal challenges

Four new macaques were then administered through a gastric tube 5 ml of a vaccine comprising iSIV and BCG. Vaccine administration was preceded and followed by the injection of 15 ml of 0.1 M sodium bicarbonate in the macaques gastric tube. Booster vaccinations with the same preparations were repeated at days 30 and 60. Four control animals received BCG alone according to the same protocol. An intrarectal viral challenge (100,000 TCID_50_ of SIVmac239) was then given to the eight animals at day 90. The four control animals showed a typical primary infection with a plasma viral peak between days 10 and 14 post-challenge. In strong contrast, the four animals that received the vaccine were sterilely protected as shown by their plasma SIV RNA and PBMC SIV DNA loads, which remained undetectable from day 1 to 60 (Figures 1F,G).

In light of these unexpected mucosal vaccination successes, we tested the SIV-specific antibody and cellular responses of vaccinated macaques. Surprisingly again, SIV-specific antibodies responses in intravaginally or intragastrically vaccinated macaques were undetectable or extremely weak while all control monkeys raised anti-SIV antibodies and became seropositive. SIV Gag p27-specific interferon γ-releasing T-cell responses were also undetectable by ELISPOT in vaccinated macaques (data not shown).

#### *Ex vivo* antiviral activity of CD8+ T-lymphocytes in vaccinated macaques

**Figure 2:**
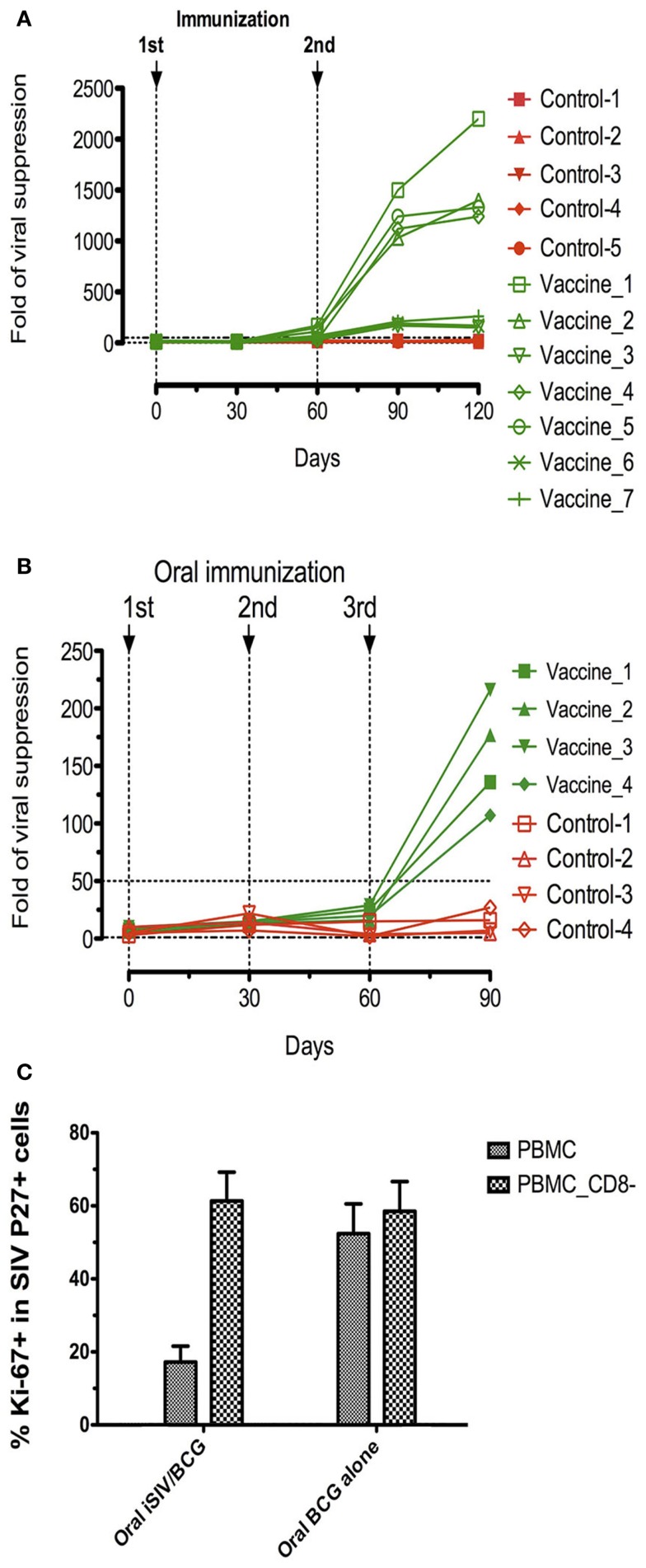
***Ex vivo* antiviral activity and suppression of SIV positive CD4+ T-cell activation generated by vaccine-induced CD8^+^ T-cells of rhesus macaques immunized with inactivated SIV and BCG**. **(A,B)** *Ex vivo* antiviral activity generated by vaccine-induced CD8^+^ T-cells in seven rhesus macaques intravaginally immunized with inactivated SIV and BCG and five control macaques **(A)** and in four rhesus macaques intragastrically immunized with inactivated SIV and BCG and four control macaques **(B)**; **(C)** percentage (mean ± SD) of the activation marker Ki-67 within SIV Gag p27^+^ cells on the gated CD4^+^ T-cells in AT-2 SIV-pulsed PBMC (depleted or not of CD8^+^ T-cells) taken from animals orally immunized with inactivated SIV plus BCG or those immunized with BCG alone.

This surprising immunovirological picture, characterized by a sterile immunity after intrarectal challenge, or complete virus replication control after intravenous challenge in the majority of mucosally vaccinated animals, in the absence of SIV-specific humoral and cellular immune responses, prompted us to examine whether SIV-specific non-conventional cellular responses could exist. For that purpose, CD4^+^ and CD8^+^ T-cells were purified from fresh PBMC of vaccinated or control animals. CD4^+^ T-cells were then acutely infected with SIVmac239 and CD4^+^ T-cells and CD4^+^ plus CD8^+^ T-cells were stimulated overnight with SEB plus anti-CD3 and anti-CD28 antibodies and cultivated for 5 days. The antiviral activity of the CD8^+^ T-cells was expressed as the ratio of SIV RNA concentration in the supernatants of SIV-infected CD4^+^ T-cell cultures in the absence of CD8^+^ T-cells over the SIV RNA concentration in corresponding cultures in the presence of CD8^+^ T-cells [for details, see “Method” in Ref. (23)]. Results of these assays showed that CD8^+^ T-cells from the four out of seven intravaginally vaccinated animals that were shown later to be protected against intrarectal challenge, showed a strong antiviral activity (ratio over 100), whereas CD8^+^ T-cells from the three animals, which were later shown not to be protected against SIV infection as well as CD8^+^ T-cells from those which were not vaccinated had baseline levels of antiviral activity (ratio <50) (Figure 2A). Similarly, in the four orally vaccinated macaques, which were all found to be protected from an intrarectal challenge some weeks later, the antiviral activity was over 100 (Figure 2B). Altogether, in this group of vaccinated macaques, we observed a complete correlation between the pre-challenge *ex vivo* antiviral activity of CD8^+^ T-cells and the protection observed after SIV challenge suggesting that the CD8^+^ T-lymphocytes antiviral activity of vaccinated macaques is an excellent predictive marker of protection against SIV challenge.

#### Suppression of activation of SIV-specific CD4^+^ T-cells by vaccine-induced CD8^+^ T-cells in vaccinated macaques

Since, the initiation of replication of SIV in macaques (or HIV in humans) *in vivo*, as well as SIV (or HIV)-specific antibody and CTL induction needs SIV (or HIV)-specific CD4^+^ T-cell activation (11), we suspected that the suppression of both SIV replication and SIV-specific immune responses observed after mucosal vaccination with iSIV plus BCG could be the consequence of the suppression of SIV-specific CD4^+^ T-cell activation [for details on the method of CD4^+^ T-cell activation suppression, see “Method” in Ref. (23)]. It was therefore reassuring to observe that SIV-positive CD4^+^ T-cell activation was suppressed in the PBMC of vaccinated macaques while remaining at the high level observed in control samples when CD8^+^ T-cells were omitted from the test sample (Figure 2C).

Altogether, the intravaginal co-administration of BCG and inactivated SIV protected four out of six monkeys from an intravenous SIVmac239 challenge and four out of seven monkeys from an intrarectal challenge while the intragastric administration of the same vaccine preparation protected four out of four macaques from an intrarectal challenge. Long-term protection (28 months) associated with this new type of vaccine was maintained after repeated intravenous and intrarectal challenges in the three animals where it was tested. In contrast, infection was observed in all control animals. In the four out of six macaques protected from an intravenous challenge, viral replication was completely controlled but PBMC chronically harbored the provirus; in contrast, in the 8 out 11 macaques protected from an intrarectal challenge, plasma SIV RNA and PBMC and lymph node cellular SIV DNA were both undetectable suggesting that SIV infection was blocked at entry by mucosal immunity or eradicated post-entry by systemic immunity. Surprisingly, we did not observe any SIV-specific antibodies or interferon γ-producing T-cells in the macaques that were shown to be protected after intrarectal challenge. Moreover, CD8^+^ T-cells isolated from PBMCs of the same macaques strongly suppressed the replication of virus in acutely infected autologous CD4^+^ T-cells *ex vivo*.

### Oral (intragastric) vaccination with inactivated SIV and *Lactobacillus plantarum*

#### Confirmatory studies and new information

Results observed after mucosal vaccination with BCG and iSIV were very striking, particularly because BCG, an anti-tuberculosis vaccine given worldwide for more than 90 years to more than a billion infants and children has never been even suspected to induce anergy/unresponsiveness or oral tolerance, either to its own constituents or to those of an immunogen given together. In view of these unexpected results, we decided to test an oral vaccine including iSIV and a bacterial adjuvant such as a *Lactobacillus* strain, a non-pathogenic intestinal commensal bacterium that had previously been suggested to potentially favor immune tolerance (30–32). We started by exploring the activity of an oral vaccine made of iSIV and LP (33). A group of eight macaques was immunized via the intragastric route with iSIV formulated with LP while eight control animals received LP only (four macaques) or iSIV only (four macaques). Results of this study (23) confirmed and extended several immunological and virological features already observed in iSIV + BCG-vaccinated macaques, viz: (1) both SIV-specific antibodies and interferon γ-secreting T-cells upon SIV Gag p27 stimulation were suppressed in iSIV + LP-vaccinated macaques; (2) SIV-positive CD4^+^ T-cell activation and SIV replication in infected CD4^+^ T-cells were also suppressed (through a non-lytic process) by the CD8^+^ T-cells of iSIV + LP-vaccinated macaques; moreover, *ex vivo*, suppression of CD4^+^ T-cell activation as well as virus replication suppression did not occur when CD8^+^ T-cells were omitted from the test samples; (3) extending our previous work, we also showed that CD8^+^ T-cells isolated from the PBMC of vaccinated macaques, when added to the test samples at a time where SIV-specific CD4^+^ T-cells activation was already established, no longer inhibited viral replication; this new information suggested a link of causality between the suppression of SIV-specific CD4^+^ T-cell activation and the suppression of viral replication by CD8^+^ T-cells. We further demonstrated that CD8^+^ T-cell-mediated antiviral activity required cell-to-cell contact and that vaccine-induced CD8^+^ T-cells operated through a non-classical MHC-restriction mechanism that was dependent on MHC-IB/E. We also showed that classical Tregs (CD4+CD25+FoxP3+) had no role in this suppression process; (4) finally, we demonstrated that, similar to iSIV + BCG-vaccinated macaques, the eight iSIV + LP-vaccinated macaques orally were sterilely protected from high dose intrarectal challenge (100,000 TCID_50_) of SIVmac239 (without any detectable level of plasma SIV RNA or PBMC proviral DNA) while the eight control animals were infected. This sterile protection was fully confirmed by the results of a second intrarectal challenge performed in four out of eight vaccinated macaques. We also showed the same virological picture as that observed in iSIV + BCG-vaccinated macaques in the four out of eight macaques, which were rechallenged via the intravenous route; a slight peak of SIV replication (≤200 DNA copies/million PBMC and 200 SIV copies/ml of plasma) was observed at day 10 post-challenge, but by day 30, plasma SIV RNA loads had dropped back to undetectable level (≤10 copies/ml) while PBMC SIV DNA were ≤10 copies/million cells showing the latent presence of SIV proviral DNA but the absence of virus replication *in vivo*. Finally, we showed that the eight iSIVmac239 + LP-vaccinated macaques (the already four intrarectally rechallenged and the already four intravenously rechallenged ones) remained fully protected against an heterologous intrarectal challenge with 100,000 TCID_50_ of the antigenically distinct SIVB670, which suggested that the vaccine was cross-protective, presumably through preventing the activation of CD4^+^ T-cells infected by another SIV strain [see Figure 4 in Ref. (23)]. Results of oral vaccinations with iSIV + BCG and iSIV + LP are summarized in Table 1.

**Table 1:** **Summary of immunovirological results observed in orally vaccinated monkeys**.

Intragastric immunization	iSIV	iSIV + BCG	iSIV + LP
Production of anti-SIV antibodies	Yes	Suppressed	Suppressed
Induction of SIV-specific IFNγ-releasing T-cells	Yes	Suppressed	Suppressed
Proliferation of CD4^+^ T-cells toward SIV antigens	NT*	NT	Suppressed
Activation of SIV^+^CD4^+^ T-cells	Yes	Suppressed	Suppressed
Ex-vivo suppressive CD8^+^ Tregs	No	Yes	Yes
Sterile protection after intrarectal homologous SIV challenge	No	Yes	Yes
Sterile protection after intrarectal heterologous SIV challenge	No	NT	Yes
Absence of SIV replication after intravenous SIV challenge	No	Yes	Yes

*NT, not tested.

#### Role of CD8^+^ T-cells in protecting macaques against intrarectal SIV challenge

To better identify the *in vivo* role of CD8^+^ T-cells in the macaques protection, the four macaques sterilely protected after three intrarectal challenge were given a fourth intrarectal challenge with 100,000 TCID_50_ of SIV239; these four macaques were at the same time depleted of their CD8^+^ T-cells from peripheral blood and lymphoid organs by a cytotoxic anti-CD8 antibody [see “Method” in Ref. (23)]. At the nadir of CD8^+^ T-cell count (day 15), plasma viral loads of the four animals peaked around 10^4^–10^6^ RNA copies/ml and 10^3^–10^4^ copies SIV DNA/10^6^ PBMC, respectively, and all animals became infected and SIV-seropositive. However, by weeks 4–7, when the CD8^+^ T-cell levels of the four monkeys had recovered to almost normal, plasma SIV RNA and PBMC and lymph node SIV DNA dropped to baseline levels. This *in vivo* experiment demonstrated the central role of CD8^+^ T-cells in immunized animals to prevent initial infection at the mucosal barrier. When CD8^+^ T-cells were depleted at the moment of the intrarectal challenge, the virus replicated freely in lymphoid organs; but as soon as the vaccine-induced CD8^+^ T-cell population recovered, they again strongly controlled viral replication. Interestingly, recovered animals now contained SIV DNA in target cells, but without obvious viral replication (23).

#### Longevity of the vaccine-induced protection

**Figure 3:**
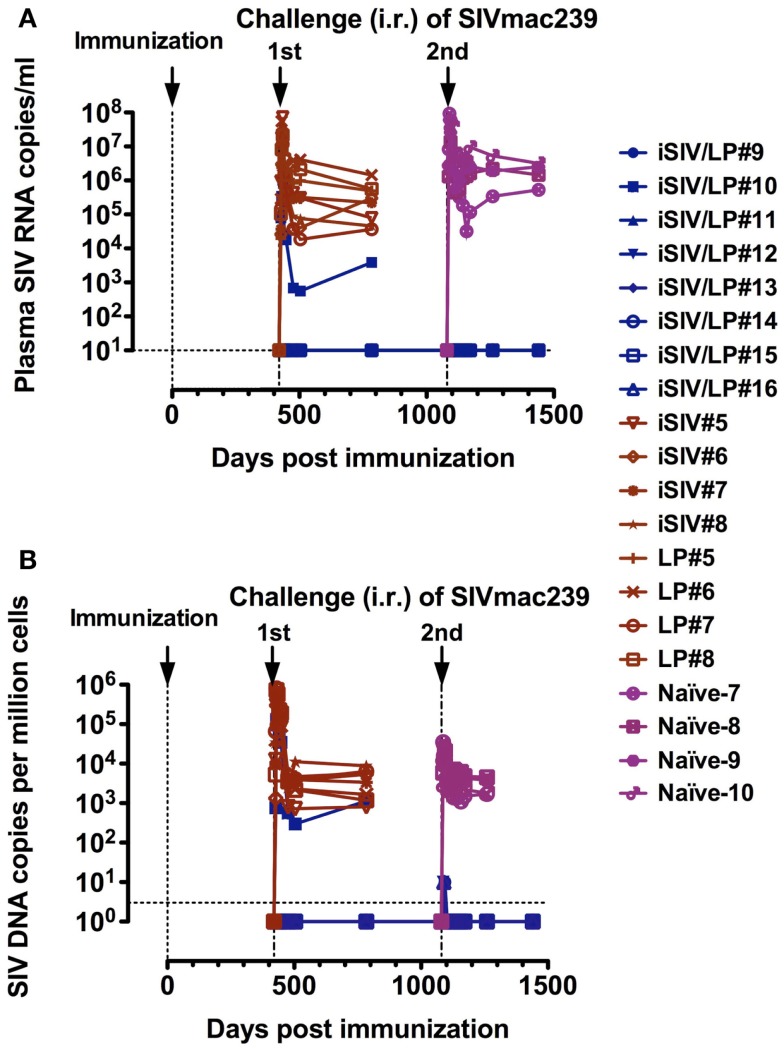
**Long-term follow-up of eight macaques intragastrically immunized with iSIV and LP and eight control monkeys immunized with iSIV alone (four macaques) or LP alone (four macaques)**. **(A,B)** Plasma viral loads **(A)** and PBMC proviral loads **(B)** of eight macaques submitted to two successive intrarectal challenges performed at 14 and 36 months post-immunization with SIVmac239 (100,000 TCID50).

To study the longevity of the vaccine protection, we immunized a new group of eight macaques with iSIV/LP in parallel with eight control macaques (four with iSIV and four with LP). We observed that seven of the eight animals that received the vaccine maintained a high *ex vivo* antiviral activity ratio (≥200) for up to 14 months while in one vaccinated macaque the antiviral activity decreased from month 12 to the baseline ratio (<100) seen in the eight control animals treated with iSIV or LP alone. By 14 months post-immunization, all 16 animals (vaccinated and controls) were challenged intrarectally with 100,000 TCID_50_ of SIVmac239. Seven out of the eight iSIV/LP-immunized animals had a sterile immunity as indicated by the absence of any SIV RNA and DNA emergence in plasma, PBMC, rectal mucosa, or pelvic lymph nodes lymphocytes. Interestingly, the one vaccinated macaque, which lost its *ex vivo* antiviral activity became fully infected in the same manner as the eight control monkeys [see Figure 5 in Ref. (23); Figures 3A,B]. Thus, the evolution of the *ex vivo* antiviral activity of the eight vaccinated monkeys allowed us to predict, from day 360 post-immunization (i.e., 60 days before their challenge) the one out of eight monkeys that would not be protected. To control further the long-term efficacy of our vaccine, the seven protected macaques were intrarectally rechallenged by 36 months after vaccination; by 48 months post vaccination, we showed that all of them remained fully protected from infection (Figures 3A,B); importantly, we controlled that the repetition of viral challenges had no role in the long-term protection of the vaccinated macaques since their CD8^+^ T-cells conserved the same high level of *ex vivo* antiviral activity before as well as after being rechallenged (data not shown).

### Oral (intragastric) vaccination with inactivated SIV and *Lactobacillus rhamnosus*

**Figure 4:**
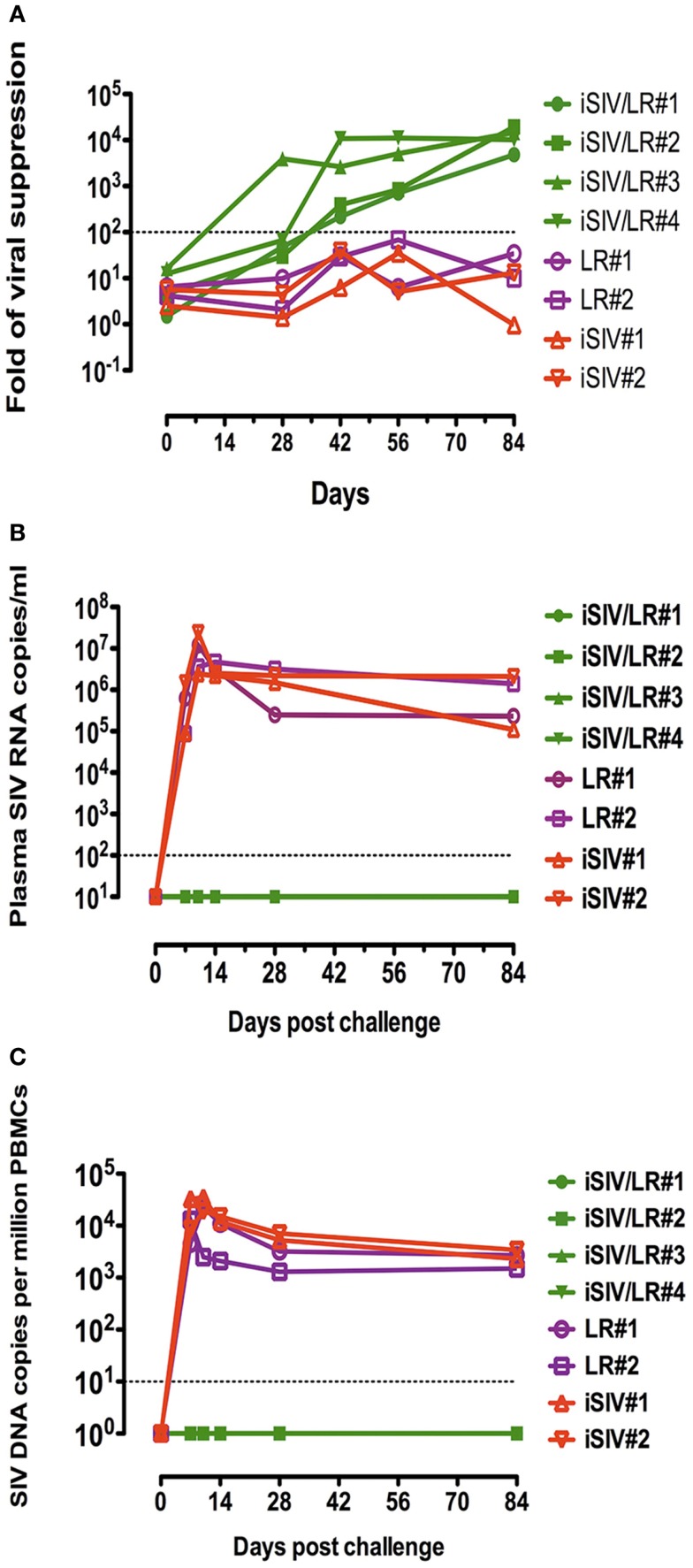
***Ex vivo* antiviral activity induced by CD8^+^ T-cells of four macaques intragastrically immunized with iSIV plus LR and viral loads of the four macaques following an intrarectal challenge**. **(A)** *Ex vivo* antiviral activity induced by CD8^+^ T-cells of four macaques intragastrically immunized with iSIV and LR and four control monkeys immunized with iSIV alone or LR alone; **(B,C)** plasma viral loads **(B)** and PBMC proviral loads **(C)** following an intrarectal challenge performed intrarectally with SIVmac239 (100,000 TCID50) at day 90 post-immunization.

Because the *Lactobacillus plantarum* strain ATCC8014 used to vaccinate macaques along with iSIV had never been used in humans, we tested the common probiotic strain *Lactobacillus rhamnosus* (LR) as bacterial adjuvant (34). Four macaques were vaccinated orally with iSIV and LR; at the same time, four control macaques received LR only (two animals) and iSIV only (two animals). All macaques were vaccinated according to the protocol used for iSIV + LP vaccination. The *ex vivo* antiviral activity following vaccination increased progressively to reach a ratio >200 in all vaccinated macaques by 2 months post vaccination (Figure 4A).

Moreover, all vaccinated animals were sterilely protected from an intrarectal challenge (100,000 TCID_50_ of SIVmac239) performed 12 weeks post vaccination (Figures 4B,C).

In summary, in the macaque model, we have demonstrated the prevention of SIV infection by inducing immunological tolerance against the infectious agent. The administration of iSIVmac239 with bacterial adjuvants such as BCG, LP, or LR, stimulated macaques to develop a thus far unrecognized type of SIV-specific immune tolerance characterized by the activation of a previously unrecognized population of non-cytolytic MHCIb/E-restricted CD8^+^ T-regulatory cells that have the apparent ability to suppress the activation of SIV-positive CD4^+^ T-cells. This suppression resulted in the blocking of SIV reverse transcription in CD4^+^ T-cells thereby preventing the initial burst of virus replication and thus protecting macaques from infection. Out of 24 intragastrically vaccinated macaques challenged with a high dose of SIVmac239 or the heterologous strain SIVB670 3 to 14 months later via the intrarectal route, 23 were sterilely protected for up to 48 months while all control macaques became infected.

## Discussion

We have identified three areas of research that will be required to establish the mechanisms involved in the observed protection.

### Characteristics of vaccine-induced CD8^+^ T-regulatory cells

The suppressive non-cytolytic MHC-IB/E-restricted CD8^+^ T-cells identified in the present study represent a new class of CD8^+^ T-regulatory cells (CD8^+^ Tregs) that has not been described previously in the context of any antiviral vaccination or in SIV or HIV infection. Thus far, the only T-regulatory cells described in HIV infection were classical Tregs (CD25^+^FoxP3^+^CD4^+^ T-cells) known to be involved in some models of immune tolerance (35). Their role in HIV infection is not clearly understood; on the one hand, the expansion of Tregs has been shown to be associated with the suppression of HIV-specific CD4^+^ T-cell responses and disease progression (36, 37) while on the other hand, Tregs have been associated with protection from productive infection, CD4^+^ T-cell activation and disease progressions in both humans (38, 39) and non-human primates (40). In the present study, the *ex vivo* removal of CD25^+^FoxP3^+^CD4^+^ T-cells by an anti-CD25 antibody did not modify either the suppression of CD4^+^ T-cell activation or that of viral replication. Except for the fact that they are non-cytolytic, the CD8^+^ Tregs observed here resemble those which targeted and eliminated abnormally activated antigen-specific CD4^+^ T-helper cells in the mouse model (41, 42) where the inhibitory interaction depends on recognition of surface Qa-1, corresponding to MHC-IB/E in macaques and to HLA-E in humans (43), expressed by aberrantly activated target cells (44, 45). Similar CD8^+^ Tregs have also been implicated in the control of autoimmune type 1 diabetes in humans (46). Interestingly, we also found CD8^+^ Tregs with the same characteristics in human elite controllers, a small percentage of HIV-infected patients (<1%) who have naturally long-term undetectable viral loads but harbor the virus in their target cells (Wei Lu and Jean-Marie Andrieu, manuscript in preparation). So far, we have not been able to identify the CD antigens specifically associated with these CD8^+^ Tregs and their phenotypic and molecular characteristics require further studies.

### Role of CD8^+^ T-regulatory cells

Because vaccinated macaques challenged via the intrarectal route were negative for both SIV RNA in plasma and proviral SIV DNA in PBMC, our results suggest that virus infection was arrested before nuclear integration. In quiescent CD4^+^ T-cells, virus penetration is followed within 2 h by the presentation at the plasma membrane of Gag/Pol and other protein epitopes derived from incoming virions (47) while Env and Nef protein presentation requires *de novo* synthesis (48). The subsequent phases of the infectious process including reverse transcription and proviral DNA integration, develop very inefficiently in quiescent CD4^+^ T-cells but very efficiently in activated CD4^+^ T-cells (49–51). This is in keeping with the notion that the early activation of a small founder population of infected CD4^+^ T-cells at the portal of entry is required for the local expansion and establishment of systemic infection (52, 53). This early event potentially gives our vaccine-induced CD8^+^ T-cells the opportunity to arrest the infectious process before proviral DNA integration. We observed that the withdrawal of our vaccine-induced CD8^+^ T-cells from PBMC cultures before CD4^+^ T-cell activation, allowed CD4^+^ T-cells to become activated and thereby viral replication to proceed. Similarly, *in vivo* depletion of the CD8^+^ T-cells performed at the time of intrarectal challenge allowed CD4^+^ T-cells to be activated and the virus to replicate. Such replication, however, was brought under control as the novel CD8^+^ T-cells recovered in lymphoid organs and the blood stream. We showed in *ex vivo* cultures that the inhibition of viral replication was directly attributable to this new class of CD8^+^ Tregs. This is also highly likely to be the case *in vivo* although direct evidence for this is lacking since we depleted the whole CD8^+^ T-cell population rather than specifically the Treg subset. In monkeys intravenously challenged, the transient peak of viral replication followed by the residual presence of cellular SIV DNA suggested that infectious SIV particles that entered the body by this route encountered activated CD4^+^ T-cells, probably in secondary lymphoid organs, which allowed the virus to complete the first cycles of replication. However, it cannot be excluded that non-activated (quiescent) CD4^+^ T-cells, which were penetrated by newly released virions, were prevented from becoming activated by the vaccine-generated CD8^+^ Treg. An in-depth understanding of the results presented here will require extensive exploratory studies of cellular and molecular immunovirology.

### Induction of SIV-specific CD8^+^ T-regulatory cells

This remains the most mysterious unanswered question. When iSIV, a particulate immunogen was administered alone via the intragastric route, it stimulated the intestinal immune system to induce the activation and proliferation of SIV-CD4^+^ T-cell as well as the generation of SIV-specific antibodies and interferon γ-producing cells according to the classical vaccine paradigm. However, this iSIV-only vaccination did not induce any *in vitro* or *in vivo* viral suppression nor any protection in challenged macaques. On the other hand, a soluble protein such as SIV P55, when intragastrically administered in its own, did not induce any immunological response nor any challenge protection (data not shown). In surprising contrast, when iSIV was administered along with BCG, or LP or LR, the reaction of the macaque intestinal immune was totally different. SIV-CD4^+^ T-cells proliferation and activation were abolished (through the contact between the TCRαβ of the vaccine-induced CD8^+^ T-regulatory cells and the MHCIb/E of SIV positive CD4^+^ T-cells). A first consequence of this vaccine-induced SIV-positive CD4^+^ T-cell unresponsiveness/anergy is most likely the suppression of any help to the induction of SIV-specific antibody- and interferon γ-producing cells; the observation of such an antigen-specific immune tolerance opens a vast array of research on the suppressive arm of the immune system and its potential manipulation in human and veterinary medicine. The other consequence of this vaccine-induced SIV-positive CD4^+^ T-cell unresponsiveness/anergy is of unique importance for vaccinologists since it is responsible for the suppression of *in vitro* and *in vivo* SIV reverse transcription and for the sterile protection of macaques against SIV challenge, bringing the experimental confirmation of our initial suspicion that suppression of CD4^+^ T-cell activation is able to suppress viral replication (9).

The mechanism of this extraordinary immunological switch is so far unknown. BCG itself has never been suspected to possess tolerogenic properties. Only in Brazil, have infants been given BCG via the oral route (over a period of 50 years from the 20s to the 70s) and Brazilian scientists found that oral BCG protected against childhood tuberculosis, meningitis, and miliary disease with the same efficacy as intradermic BCG (54). However, orally vaccinated infants remained generally unresponsive/anergic to the post vaccination tuberculin test, which became generally positive after classical intradermic vaccination (55). In the same line, oral BCG vaccination inhibited delayed-type hypersensitivity to purified protein derivative (while at the same time it induced interferon γ-secreting T-cells upon *Mycobacterium* antigens stimulation) (56). Moreover, it has also been observed that when the central nervous system of mice was infected with BCG, the mice were able to overcome experimental autoimmune encephalomyelitis, a classical mice model of autoimmune disease (57). The possibility that BCG may possess certain unexplored tolerogenic properties seems worthy of further research inasmuch as CD8^+^ Tregs have been very recently shown to dominate suppressive phenotype and function after BCG activation of human cells (58).

On the other hand, there is some experimental evidence suggesting that LP and LR may have some tolerogenic properties (30–32), which may be involved in their probiotic ability to fight human inflammatory bowel disease (59). However, the mechanism of induction of such a tolerance seems to be via the generation of classical CD25^+^FoxP3^+^CD4^+^ Tregs (60, 61) and not through the intervention of CD8^+^ Tregs.

It must be mentioned that recombinant BCG expressing viral antigens including HIV and SIV proteins has been tested via the mucosal route in non-human primates. These recombinant BCGs induced IgA against BCG-expressed viral proteins but were never found to induce any humoral or cellular sign of virus-induced immune tolerance (62). Similarly, lactobacilli and lactococci engineered to express antigenic proteins also including SIV antigens have been mucosally administered to different animal models including non-human primates; whatever the antigen expressed, the immunological result of the mucosal administration was always the production of specific of antigenic protein-directed antibodies and particularly of IgA by the intestinal immune system (63).

Our results demonstrate that an oral vaccine comprising iSIV, a particulate immunogen adjuvanted by BCG, LP, or LR administered intragastrically to a large number of macaques stimulated their intestinal immune system and generated a new class of CD8^+^ Tregs that suppressed the activation of mucosal and systemic SIV-positive CD4^+^ T-cells. This suppression of CD4^+^ T-cell activation inhibited viral replication and thereby prevented SIV infection in the macaque model. The protective effect of this innovative immunization regimen against SIV challenge, together with the identification of a correlate of protection *ex vivo*, is quite striking. Given that SIV and HIV require activated CD4^+^ T-cells in which to replicate, this tolerogenic vaccine approach may offer an exciting new avenue in preventive HIV vaccine research. We are preparing a randomized phase 1 trial where a small population of non-at-risk volunteers will receive the vaccine or a placebo; the vaccination success will be defined by the increase of the *ex vivo* antiviral activity of CD8^+^ T-cells (ratio >200) in the vaccinated group while it will remain low and stable (ratio <100) in the placebo group. On the other hand, the *de novo* induction of this class of CD8^+^ Tregs could potentially be used therapeutically to maintain HIV replication suppression in infected patients in whom antiviral treatments have been interrupted. We are now preparing a phase 1 trial where infected volunteers with undetectable viral loads under antiviral therapy will be orally vaccinated with heat-inactivated HIV and LR; the antiviral treatment of infected volunteers will be suspended at week 24. The success of this trial will be ascertained by the increase of *ex vivo* antiviral activity of CD8^+^ T-cells (ratio >200) by 8 weeks post vaccination and by permanent undetectable plasma viral loads from week 24 (antiviral treatment withdrawal). The new tolerogenic vaccine paradigm described in the present study, beside its potential use in the HIV vaccine field, could potentially be exploited in the management of a wide range of immune disorders and could uncover so far unrecognized immunological mechanisms.

## Conflict of Interest Statement

Jean-Marie Andrieu and Wei Lu have received grants from and are shareholders of Biovaxim Ltd. The other co-authors report no conflicts of interest.

## References

[1] Plotkin SA Correlates of protection induced by vaccination. Clin Vaccine Immunol (2010) 7:1055–65 10.1128/CVI.00131-10 20463105 PMC2897268

[2] Barré-Sinoussi F Chermann JC Rey F Nugeyre MT Chamaret S Gruest J Isolation of a T-lymphotropic retrovirus from a patient at risk for acquired immune deficiency syndrome (AIDS). Science (1983) 220(4599):868–71 10.1126/science.6189183 6189183

[3] Esparza J A brief history of the global effort to develop a preventive HIV vaccine. Vaccine (2013) 31(35):3502–18 10.1016/j.vaccine.2013.05.018 23707164

[4] Rerks-Ngarm S Pitisuttithum P Nitayaphan S Kaewkungwal J Chiu J Paris R Vaccination with ALVAC and AIDSVAX to prevent HIV-1 infection in Thailand. N Engl J Med (2009) 361:2209–20 10.1056/NEJMoa0908492 19843557

[5] Duerr A Huang Y Buchbinder S Coombs RW Sanchez J del Rio C Extended follow-up confirms early vaccine-enhanced risk of HIV acquisition and demonstrates waning effect over time among participants in a randomized trial of recombinant adenovirus HIV vaccine (Step Study). J Infect Dis (2012) 206(2):258–66 10.1093/infdis/jis342 22561365 PMC3490694

[6] Gray GE Moodie Z Metch B Gilbert PB Bekker LG Churchyard G Recombinant adenovirus type 5 HIV gag/pol/nef vaccine in South Africa: unblinded, long-term follow-up of the phase 2b HVTN 503/Phambili study. Lancet Infect Dis (2014) 14(5):388–96 10.1016/S1473-3099(14)70020-9 24560541 PMC4174314

[7] Hammer SM Sobieszczyk ME Janes H Karuna ST Mulligan MJ Grove D Efficacy trial of a DNA/rAd5 HIV-1 preventive vaccine. N Engl J Med (2013) 369(22):2083–92 10.1056/NEJMoa1310566 24099601 PMC4030634

[8] Fauci AS Marovich MA Dieffenbach CW Hunter E Buchbinder SP Immunology. Immune activation with HIV vaccines. Science (2014) 344(6179):49–51 10.1126/science.1250672 24700849 PMC4414116

[9] Andrieu JM Even P Venet A AIDS and related syndromes as a viral-induced autoimmune disease of the immune system: an anti-MHC II disorder. Therapeutic implications. AIDS Res (1986) 2(3):163–74 10.1089/aid.1.1986.2.163 3489470

[10] Paiardini M Müller-Trutwin M HIV-associated chronic immune activation. Immunol Rev (2013) 254(1):78–101 10.1111/imr.12079 23772616 PMC3729961

[11] Klatt NR Chomont N Douek DC Deeks SG Immune activation and HIV persistence: implications for curative approaches to HIV infection. Immunol Rev (2013) 254(1):326–42 10.1111/imr.12065 23772629 PMC3694608

[12] Andrieu JM Even P Venet A Tourani JM Stern M Lowenstein W Effects of cyclosporine on T-cell subsets in human immunodeficiency virus disease. Clin Immunol Immunopathol (1988) 46:181–98 10.1016/0090-1229(88)90071-2 3258211

[13] Levy R Jais JP Tourani JM Even P Andrieu JM Long-term follow-up of HIV positive asymptomatic patients having received cyclosporine A. Adv Exp Med Biol (1995) 374:229–34 10.1007/978-1-4615-1995-9_20 7572396

[14] Andrieu JM Lu W Levy R Sustained increases in CD4 cell counts in asymptomatic human immunodeficiency virus type 1-seropositive patients treated with prednisolone for 1 year. J Infect Dis (1995) 171(3):523–30 10.1093/infdis/171.3.523 7876597

[15] Andrieu JM Lu W Long-term clinical, immunologic and virologic impact of glucocorticoids on the chronic phase of HIV infection. BMC Med (2004) 2:17 10.1186/1741-7015-2-17 15128452 PMC411065

[16] Lu W Salerno-Goncalves R Yuan J Sylvie D Han DS Andrieu JM Glucocorticoids rescue CD4+ T-lymphocytes from activation-induced apoptosis triggered by HIV-1: implications for pathogenesis & therapy. AIDS (1995) 9:35–42 10.1097/00002030-199501000-00005 7893439

[17] Lu W Andrieu JM In vitro human immunodeficiency virus eradication by autologous CD8(+) T-cells expanded with inactivated-virus-pulsed dendritic cells. J Virol (2001) 75(19):8949–56 10.1128/JVI.75.19.8949-8956.2001 11533158 PMC114463

[18] Lu W Wu X Lu Y Guo W Andrieu JM Therapeutic dendritic-cell vaccine for simian AIDS. Nat Med (2003) 9:27–32 10.1038/nm806 12496959

[19] Lu W Arraes LC Ferreira WT Andrieu JM Therapeutic dendritic-cell vaccine for chronic HIV-1 infection. Nat Med (2004) 10:1359–65 10.1038/nm1147 15568033

[20] García F Climent N Guardo AC Gil C León A Autran B A dendritic cell-based vaccine elicits T cell responses associated with control of HIV-1 replication. Sci Transl Med (2013) 5(166):166ra2 10.1126/scitranslmed.3004682 23283367

[21] Chen S Lai C Wu X Lu Y Han D Guo W Variability of bio-clinical parameters in Chinese-origin Rhesus macaques infected with simian immunodeficiency virus: a nonhuman primate AIDS model. PLoS One (2011) 6:e23177 10.1371/journal.pone.0023177 21850259 PMC3151272

[22] Stahl-Hennig C Suh YS Park KS Sauermann U Kim KS Ahn S Immunogenicity of a DNA prime and recombinant adenovirus boost regime significantly varies between rhesus macaques of Chinese and Indian origins. J Med Primatol (2007) 36:195–205 10.1111/j.1600-0684.2007.00237.x 17669208

[23] Lu W Chen S Lai C Guo W Fu L Andrieu JM Induction of CD8+ regulatory T-cells protects macaques against SIV challenge. Cell Rep (2012) 2(6):1736–46 10.1016/j.celrep.2012.11.016 23260669

[24] Mangtani P Abubakar I Ariti C Beynon R Pimpin L Fine PE Protection by BCG vaccine against tuberculosis: a systematic review of randomized controlled trials. Clin Infect Dis (2014) 58(4):470–80 10.1093/cid/cit790 24336911

[25] Ramoner R Rieser C Herold M Klocker H Bartsch G Stenzl A Activation of human dendritic cells by bacillus Calmette-Guerin. J Urol (1998) 159(5):1488–92 10.1097/00005392-199805000-00021 9554339

[26] Demangel C Britton WJ Interaction of dendritic cells with mycobacteria: where the action starts. Immunol Cell Biol (2000) 78:318–24 10.1046/j.1440-1711.2000.00935.x 10947855

[27] Gandhi NM Morales A Lamm DL Bacillus Calmette-Guérin immunotherapy for genitourinary cancer. BJU Int (2013) 112(3):288–97 10.1111/j.1464-410X.2012.11754.x 23517232

[28] Stewart JH IV Levine EA Role of bacillus Calmette-Guérin in the treatment of advanced melanoma. Expert Rev Anticancer Ther (2011) 11(11):1671–6 10.1586/era.11.163 22050015

[29] Benévolo-de-Andrade TC Monteiro-Maia R Cosgrove C Castello-Branco LR BCG Moreau Rio de Janeiro – an oral vaccine against tuberculosis – review. Mem Inst Oswaldo Cruz (2005) 100(5):459–65 10.1590/S0074-02762005000500002 16184220

[30] Grangette C Nutten S Palumbo E Morath S Hermann C Dewulf J Enhanced anti-inflammatory capacity of a *Lactobacillus plantarum* mutant synthesizing modified teichoic acids. Proc Natl Acad Sci USA (2005) 102:10321–6 10.1073/pnas.0504084102 15985548 PMC1177390

[31] van Baarlen P Troost FJ van Hemert S van der Meer C de Vos WM de Groot PJ Differential NF-kappa B pathways induction by *Lactobacillus plantarum* in the duodenum of healthy humans correlating with immune tolerance. Proc Natl Acad Sci U S A (2009) 106:2371–6 10.1073/pnas.0809919106 19190178 PMC2650163

[32] Baba N Samson S Bourdet-Sicard R Rubio M Sarfati M Commensal bacteria trigger a full dendritic cell maturation program that promotes the expansion of non-Tr1 suppressor T-cells. J Leukoc Biol (2008) 84:1468–76 10.1189/jlb.0108017 18511576

[33] Kleerebezem M Boekhorst J van Kranenburg R Molenaar D Kuipers OP Leer R Complete genome sequence of *Lactobacillus plantarum* WCFS1. Proc Natl Acad Sci USA (2003) 100:1990–5 10.1073/pnas.0337704100 12566566 PMC149946

[34] Nivoliez A Camares O Paquet-Gachinat M Bornes S Forestier C Veisseire P Influence of manufacturing processes on in vitro properties of the probiotic strain *Lactobacillus rhamnosus* Lcr35^®^. J Biotechnol (2012) 160(3–4):236–41 10.1016/j.jbiotec.2012.04.005 22542933

[35] Faria AM Weiner HL Oral tolerance. Immunol Rev (2005) 206:232–59 16048553 10.1111/j.0105-2896.2005.00280.x PMC3076704

[36] Aandahl EM Michaelsson J Moretto WJ Hecht FM Nixon DF Human CD4+ CD25+ regulatory T-cells control T-cell responses to human immunodeficiency virus and cytomegalovirus antigens. J Virol (2004) 78:2454–9 10.1128/JVI.78.5.2454-2459.2004 14963140 PMC369239

[37] Nilsson J Boasso A Velilla PA Zhang R Vaccari M Franchini G HIV-1-driven regulatory T-cell accumulation in lymphoid tissues is associated with disease progression in HIV/AIDS. Blood (2006) 108:3808–17 10.1182/blood-2006-05-021576 16902147 PMC1895475

[38] Card CM McLaren PJ Wachihi C Kimani J Plummer FA Fowke KR Decreased immune activation in resistance to HIV-1 infection is associated with an elevated frequency of CD4(+)CD25(+)FOXP3(+) regulatory T-cells. J Infect Dis (2009) 199:1318–22 10.1086/597801 19301980

[39] Chase AJ Yang HC Zhang H Blankson JN Siliciano RF Preservation of FoxP3+ regulatory T-cells in the peripheral blood of human immunodeficiency virus type 1-infected elite suppressors correlates with low CD4+ T-cell activation. J Virol (2008) 82:8307–15 10.1128/JVI.00520-08 18579608 PMC2519624

[40] Kornfeld C Ploquin MJ Pandrea I Faye A Onanga R Apetrei C Anti-inflammatory profiles during primary SIV infection in African green monkeys are associated with protection against AIDS. J Clin Invest (2005) 115:1082–91 10.1172/JCI200523006 15761496 PMC1062895

[41] Kim HJ Verbinnen B Tang X Lu L Cantor H Inhibition of follicular T-helper cells by CD8(+) regulatory T-cells is essential for self tolerance. Nature (2010) 467:328–32 10.1038/nature09370 20844537 PMC3395240

[42] Sarantopoulos S Lu L Cantor H Qa-1 restriction of CD8+ suppressor T-cells. J Clin Invest (2004) 114:1218–21 10.1172/JCI200423152 15520850 PMC524234

[43] Pietra G Romagnani C Manzini C Moretta L Mingari MC The emerging role of HLA-E-restricted CD8+ T-lymphocytes in the adaptive immune response to pathogens and tumors. J Biomed Biotechnol (2010) 2010:907–14 10.1155/2010/907092 20634877 PMC2896910

[44] Kim HJ Wang X Radfar S Sproule TJ Roopenian DC Cantor H CD8+ T regulatory cells express the Ly49 Class I MHC receptor and are defective in autoimmune prone B6-Yaa mice. Proc Natl Acad Sci U S A (2011) 108:2010–5 10.1073/pnas.1018974108 21233417 PMC3033298

[45] Nagarajan NA Gonzalez F Shastri N Nonclassical MHC class Ib-restricted cytotoxic T-cells monitor antigen processing in the endoplasmic reticulum. Nat Immunol (2012) 13:579–86 10.1038/ni.2282 22522492 PMC3362685

[46] Jiang H Canfield SM Gallagher M Jiang HH Jiang Y Zheng Z HLA-E-restricted regulatory CD8(+) T-cells are involved in development and control of human autoimmune type 1 diabetes. J Clin Invest (2010) 120:3641–50 10.1172/JCI43522 20877010 PMC2947239

[47] Sacha JB Chung C Rakasz EG Spencer SP Jonas AK Bean AT Gag-specific CD8+ T-lymphocytes recognize infected cells before AIDS-virus integration and viral protein expression. J Immunol (2007) 178:2746–54 10.4049/jimmunol.178.5.2746 17312117 PMC4520734

[48] Sacha JB Chung C Reed J Jonas AK Bean AT Spencer SP Pol-specific CD8+ T-cells recognize simian immunodeficiency virus-infected cells prior to Nef-mediated major histocompatibility complex class I down regulation. J Virol (2007) 81:11703–12 10.1128/JVI.00926-07 17699580 PMC2168778

[49] Korin YD Zack JA Nonproductive human immunodeficiency virus type 1 infection in nucleoside-treated G0 lymphocytes. J Virol (1999) 73:6526–32 10400748 10.1128/jvi.73.8.6526-6532.1999 PMC112735

[50] Zack JA Arrigo S Weitsman SR Go AS Haislip A Chen IS HIV-1 entry into quiescent primary lymphocytes: molecular analysis reveals a labile, latent viral structure. Cell (1990) 61:213–22 2331748 10.1016/0092-8674(90)90802-l

[51] Vatakis DN Kim S Kim N Chow SA Zack JA Human immunodeficiency virus integration efficiency and site selection in quiescent CD4+ T-cells. J Virol (2009) 83:6222–33 10.1128/JVI.00356-09 19369341 PMC2687367

[52] Haase AT Early events in sexual transmission of HIV and SIV and opportunities for interventions. Annu Rev Med (2012) 62:127–39 10.1146/annurev-med-080709-124959 21054171

[53] Zhang Z Wietgrefe SW Li Q Shore MD Duan L Reilly C Roles of substrate availability and infection of resting and activated CD4+ T-cells in transmission and acute simian immunodeficiency virus infection. Proc Natl Acad Sci U S A (2004) 101:5640–5 10.1073/pnas.0308425101 15064398 PMC397458

[54] Antas PR Castello-Branco LR New vaccines against tuberculosis: lessons learned from BCG immunization in Brazil. Trans R Soc Trop Med Hyg (2008) 102(7):628–30 10.1016/j.trstmh.2008.03.014 18440575

[55] Menzies D What does tuberculin reactivity after bacille Calmette- Guérin vaccination tell us? Clin Infect Dis (2000) 31(Suppl 3):S71–4 10.1086/314075 11010826

[56] Hoft DF Brown RM Belshe RB Mucosal bacille Calmette-Guérin vaccination of humans inhibits delayed-type hypersensitivity to purified protein derivative but induces mycobacteria-specific interferon-gamma responses. Clin Infect Dis (2000) 30(Suppl 3):S217–22 10.1086/313864 10875787

[57] Lee J Reinke EK Zozulya AL Sandor M Fabry Z *Mycobacterium bovis* bacille Calmette-Guérin infection in the CNS suppresses experimental autoimmune encephalomyelitis and Th17 responses in an IFN-gamma-independent manner. J Immunol (2008) 181(9):6201–12 10.4049/jimmunol.181.9.6201 18941210 PMC2735452

[58] Boer MC van Meijgaarden KE Joosten SA Ottenhoff TH CD8+ regulatory T-cells, and not CD4+ T-cells, dominate suppressive phenotype and function after in vitro live *Mycobacterium bovis*-BCG activation of human cells. PLoS One (2014) 9(4):e94192 10.1371/journal.pone.0094192 24714620 PMC3979753

[59] Sheil B Shanahan F O’Mahony L Probiotic effects on inflammatory bowel disease. J Nutr (2007) 137(3 Suppl 2):819S–24S 17311981 10.1093/jn/137.3.819S

[60] Smits HH Engering A van der Kleij D de Jong EC Schipper K van Capel TM Selective probiotic bacteria induce IL-10-producing regulatory T-cells in vitro by modulating dendritic cell function through dendritic cell-specific intercellular adhesion molecule 3-grabbing nonintegrin. J Allergy Clin Immunol (2005) 115(6):1260–7 10.1016/j.jaci.2005.03.036 15940144

[61] Scott CL Aumeunier AM Mowat AM Intestinal CD103+ dendritic cells: master regulators of tolerance? Trends Immunol (2011) 32(9):412–9 10.1016/j.it.2011.06.003 21816673

[62] Chapman R Chege G Shephard E Stutz H Williamson AL Recombinant *Mycobacterium bovis* BCG as an HIV vaccine vector. Review. Curr HIV Res (2010) 8(4):282–98 10.2174/157016210791208686 20353397 PMC3188323

[63] Xin KQ Hoshino Y Toda Y Igimi S Kojima Y Jounai N Immunogenicity and protective efficacy of orally administered recombinant *Lactococcus lactis* expressing surface-bound HIV Env. Blood (2003) 102(1):223–8 10.1182/blood-2003-01-0110 12649143

